# Involvement of the Calmodulin-like Protein Gene *VaCML92* in Grapevine Abiotic Stress Response and Stilbene Production

**DOI:** 10.3390/ijms242115827

**Published:** 2023-10-31

**Authors:** Olga A. Aleynova, Konstantin V. Kiselev, Andrey R. Suprun, Alexey A. Ananev, Alexandra S. Dubrovina

**Affiliations:** Laboratory of Biotechnology, Federal Scientific Center of the East Asia Terrestrial Biodiversity, Far Eastern Branch of the Russian Academy of Sciences, 690022 Vladivostok, Russiakiselev@biosoil.ru (K.V.K.);

**Keywords:** stress tolerance, calcium, CML, cold stress, secondary metabolites, resveratrol, piceid

## Abstract

Calmodulin-like proteins (CMLs) are an important family of plant calcium sensor proteins that sense and decode changes in the intracellular calcium concentration in response to environmental and developmental stimuli. Nonetheless, the specific functions of individual CML family members remain largely unknown. This study aims to explore the role of the *Vitis amurensis VaCML92* gene in the development of its high stress resistance and the production of stilbenes. The expression of *VaCML92* was sharply induced in *V. amurensis* cuttings after cold stress. The *VaCML92* gene was cloned and its role in the abiotic stress responses and stilbene production in grapevine was further investigated. The *VaCML92*-overexpressing callus cell cultures of *V. amurensis* and soil-grown plants of *Arabidopsis thaliana* exhibited enhanced tolerance to cold stress and, to a lesser extent, to the drought, while their tolerance to heat stress and high salinity was not affected. In addition, the overexpression of *VaCML92* increased stilbene production in the *V. amurensis* cell cultures by 7.8–8.7-fold. Taken together, the data indicate that the *VaCML92* gene is involved as a strong positive regulator in the rapid response to cold stress, the induction of cold stress resistance and in stilbene production in wild grapevine.

## 1. Introduction

Calcium plays a crucial role as a second messenger in plant responses to various environmental cues and in physiological processes, including various environmental stimuli, seed germination, phytohormone signaling, pollen tube elongation, post-fertilization cell polarity, apoptosis and plant senescence [1,2,3]. Plant calcium sensor proteins encompass different families, including calmodulins (CaMs), calmodulin-like proteins (CMLs), calcium-dependent protein kinases (CDPKs), calcineurin B-like proteins (CBLs) and CBL-interacting protein kinases (CIPKs) [4,5,6].

Within the calcium sensor gene families, the CML family is the plant gene family with the largest number of genes [7,8,9,10]. This gene family comprises 50 genes in *Arabidopsis* [11] and at least 54 *CML* genes in grapevine [12]. The abundance and diversity of CMLs in plants suggests their critical roles in plant survival and productivity; however, there remains a lack of a comprehensive understanding regarding the biological functions of CMLs and other types of calcium-binding proteins in plants [8]. Nevertheless, the available research indicates that plant CMLs are involved in the regulation of plant growth, pathogen defense and abiotic stress adaptation [13,14,15,16,17]. The expression of specific *CML* genes has been shown to be activated in response to pathogen infection [18] or stress hormones such as methyl jasmonate, or MeJA (AtCML39), and salicylic acid, or SA (AtCML43) [19,20].

*Vitis amurensis* Rupr., commonly known as the Amur grapevine, exhibits remarkable stress resistance and stilbene content compared to other Vitaceae species [21,22]. Therefore, this species has become an invaluable resource for studying the mechanisms underlying resistance to abiotic stress, as well as the biosynthesis of stilbenes, which are phenolic secondary metabolites known for their diverse health benefits and plant protective effects [23]. In recent research, the expression of 32 *VaCMLs* was shown to actively respond to several abiotic stresses (water deficit, high salinity, high mannitol, cold and heat stresses), exhibiting both positive and negative regulation patterns [10]. For example, the genes *VaCML44*, *VaCML61*, *VaCML86* and *VaCML89* were strongly induced under salt and cold stress. In contrast, the expression of genes such as *VaCML21*, *VaCML22* and *VaCML52* increased under heat stress. Moreover, the genes *VaCML79*, *VaCML83*, *VaCML92*, *VaCML93*, *VaCML106* and *VaCML110* exhibited increased expression levels when exposed to water deficit, mannitol-induced osmotic stress and high salinity [12].

Several studies have demonstrated that the overexpression of *CML* genes in plants enhanced their resistance to abiotic stress or contributed to plant–pathogen protection, supporting the roles of certain CMLs as positive regulators of plant abiotic and biotic stress adaptation [24,25,26,27]. For instance, CaCML13 has been found to play a positive role in enhancing pepper immunity against *Ralstonia solanacearum* infection by establishing a feedback loop with the CabZIP63 transcriptional factor [16]. The overexpression of *StCBL4* and *StCIPK2* genes individually and synergistically has been proven to increase the tolerance of potato plants to the soilborne plant pathogen fungi, *Rhizoctonia solani*, in *Nicotiana benthamiana* [28]. Recent findings indicate that the overexpression of the *VaCML21v2* and *VaCML21v4* splice variants promoted a biomass accumulation of *V. amurensis* callus cell cultures under prolonged low-temperature stress and improved the survival rates of the transgenic *A. thaliana* plants after freezing [29].

There is evidence indicating the involvement of certain *CML* members in the regulation of secondary metabolite production in plants. For instance, several grapevine *CML* genes, including *VaCML52*, *VaCML65*, *VaCML93* and *VaCML95*, were highly up-regulated in the leaves and cell cultures of *V. amurensis* when exposed to various inducers of stilbene biosynthesis, such as stress hormones (methyl jasmonate, salicylic acid), stilbene precursors, or UV-irradiation [30]. At the same time, a number of *VaCML* genes were highly activated in response to only one specific inductor of stilbene biosynthesis, such as *VaCML92* in response to UV-irradiation or *VaCML108* in response to salicylic acid [30]. Furthermore, the overexpression of the *VaCML65* gene controlled by the double CaMV 35S promoter enhanced stilbene production and the expression of stilbene biosynthesis genes in *V. amurensis* callus cell cultures [31].

Previously, a pronounced activation of *VaCML92* expression was reported in response to several abiotic stress factors, including water deficit, mannitol-induced osmotic stress, high salinity and heat stress [10] and UV-irradiation [30]. The aim of this study was to investigate and verify the potential role of *VaCML92* as a regulator of plant abiotic stress resistance and stilbene production by overexpressing the *VaCML92* gene in the *V. amurensis* callus cell cultures and *A. thaliana* plants.

## 2. Results

### 2.1. Stress Tolerance of VaCML92-Overexpressing Cell Cultures of V. amurensis

Using *Agrobacteria* with pZP plasmids [32], a total of three *VaCML92*-transgenic *V. amurensis* callus cell lines (92-1, 92-2 and 92-3) transformed using the pZP-RCS2-*VaCML92-nptII* construct to overexpress the *VaCML92* gene and the control cell line (VC) transformed using the empty pZP-RCS2-*nptII* construct were independently obtained. The *VaCML* genes and the selective marker *nptII* gene were driven by the double CaMV 35S promoters. The selected transformed *calli* exhibited uniform and vigorous growth, maintaining tissue homogeneity without differentiation on the W_B/A_ medium supplemented with 6-benzylaminopurine (BAP) and α-naphthaleneacetic acid (NAA) in the dark. The control VC *calli* exhibited similar morphological, growth and biosynthetic characteristics to the parent V7 culture.

**Figure 1:**
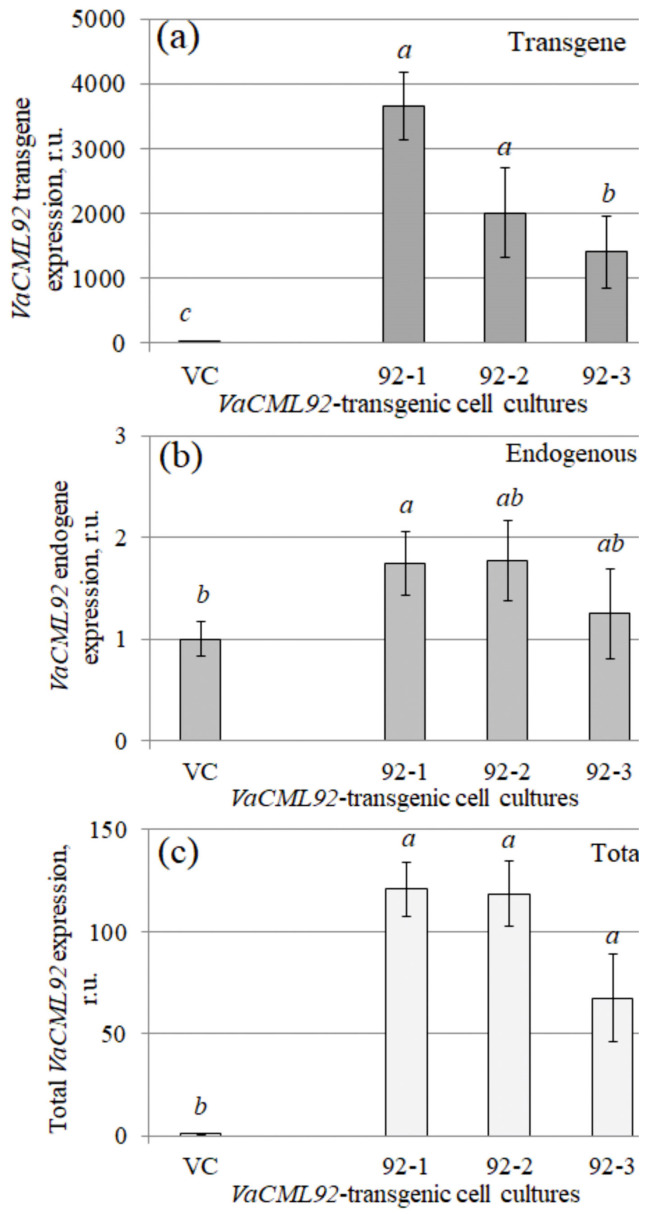
Quantification of the transgene (**a**), endogenous (**b**) and total (**c**) *VaCML92* expression in transgenic *Vitis amurensis* cell cultures performed through RT-qPCR. RNA was extracted from the vector control (VC) and *VaCML92*-transformed (91-1, 92-2 and 92-3) cell lines. Data are presented as mean ± SE. Means followed by the same letter were not different using Student’s *t*-test. *p* < 0.05 was considered to be statistically significant.

The transgenic, endogenous and total *VaCML92* expression were analyzed using different primer sets (Figure 1; Table S1). The RT-qPCR analysis revealed that the *VaCML92* transgene was actively expressed in the 92-1, 92-2 and 92-3 cell lines (Figure 1a). The highest expression of the *VaCML92* transgene was detected in the 92-1 cell line, while its expression in 92-2 and 92-3 was 1.8–2.6 times lower than in 92-1 (Figure 1a). It was then important to determine whether the overexpression of the *VaCML92* transgene affected the expression of the endogenous *VaCML92*. The data showed that the expression of endogenous *VaCML92* increased 1.8-fold in 92-1 after transformation, whereas it was not considerably affected in the 92-2 and 92-3 lines in comparison with the control VC cell line (Figure 1b). In the grapevine *calli* transformed with different *VaCDPK* genes, the expression of the corresponding endogenous *CDPK* genes was either not changed or decreased after agrobacterial transformation and overexpression of the transgene [33]. In the *VaCML92*-transformed 92-1, 92-2 and 92-3 cell lines, high levels of total *VaCML92* expression (expression of the transgene and endogenous *VaCML92*) were detected compared to the expression in the control VC culture (Figure 1c). Thus, transgenic *callus* cell lines overexpressing the *VaCML92* gene were successfully obtained and could be used to study the function of *VaCML92*.

**Figure 2:**
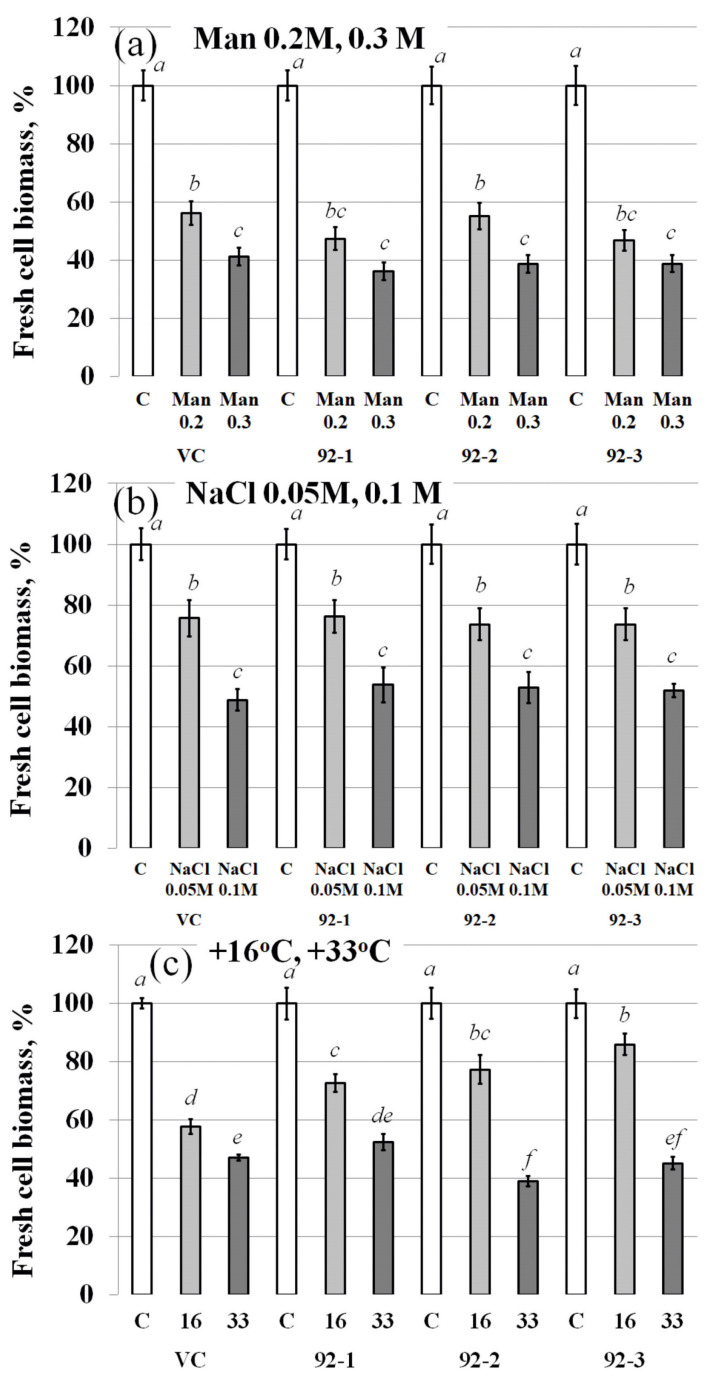
Effect of osmotic (**a**), salt (**b**), cold and heat (**c**) stress on fresh biomass accumulation of the transgenic *Vitis amurensis* cell lines overexpressing the *VaCML92* gene after 30 days of cultivation. VC—the vector control cell line of *V. amurensis*; 91-1, 92-2 and 92-3—*VaCML92*-transformed cell lines. Data are presented as mean ± SE. Means followed by the same letter were not different using Student’s *t*-test. *p* < 0.05 was considered to be statistically significant.

As it has previously been shown that *VaCML92* expression was strongly induced under water deficit, mannitol-induced osmotic stress and salt stress [10], the established *VaCML92*-overexpressing *callus* cell lines were analyzed for tolerance to major abiotic stresses (Figure 2). Surprisingly, the transgenic *calli* were not resistant to osmotic stress and high salinity (Figure 2a,b). The *VaCML92*-overexpressing cell lines. 92-1, 92-2 and 92-3, accumulated fresh biomass at the same rate as the control *calli* under mannitol- and NaCl-induced stress. All of the transgenic cell lines showed significantly better growth than the control VC culture at 16 °C (Figure 2c). Under low temperature stress conditions, the growth of all three cell lines was inhibited 1.2–1.4-fold, whereas the growth of the VC cell line was inhibited 1.7-fold (Figure 2c). Thus, the overexpression of the *VaCML92* gene had a positive effect on the cold stress resistance of the *calli*, while osmotic and salt stress resistance were not affected.

### 2.2. Stress Tolerance of VaCML92-Overexpressing Arabidopsis Plants

**Figure 3:**
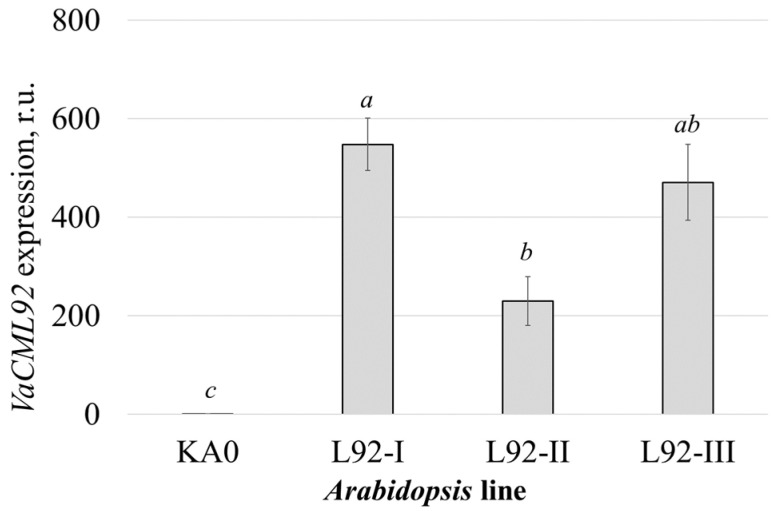
Quantification of *VaCML92* mRNAs in the transgenic *Arabidopsis thaliana* plants performed through real-time RT-qPCR. RNA was extracted from the leaves of the control plant line (KA0) and three *VaCML92*-transformed *A. thaliana* plant lines (L92-I, L92-II and L92-III). Data are presented as mean ± SE. Means followed by the same letter were not different using Student’s *t*-test. *p* < 0.05 was considered to be statistically significant.

Subsequently, using the same agrobacteria and pZP plasmids [32], a total of three homozygous *VaCML92*-transgenic lines of *Arabidopsis thaliana* plants were independently obtained using the floral dip method [34]. The highest expression of the *VaCML92* transgene was detected in the L92-I and L92-III lines, while the expression of the *VaCML92* transgene in the L92-II transgenic line was 2.1–2.4 times lower than in L92-I and L92-III (Figure 3).

**Figure 4:**
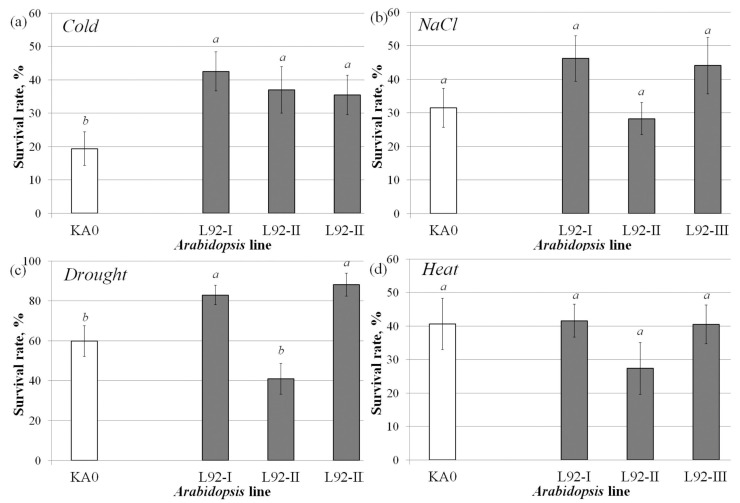
The responses to cold, salt, drought and heat stress of the control (KA0) and *VaCML92*-transgenic (L92-I, L92-II, L92-III) *Arabidopsis thaliana* plant lines. Survival rates were determined as the number of visibly green plants at the end of the experiments. Twenty plants from each line were used in each of six experiments. Data are presented as mean ± SE. Means followed by the same letter were not different using Student’s *t*-test. *p* < 0.05 was considered to be statistically significant.

The resistance of the obtained *VaCML92*-transgenic *A. thaliana* plants to cold, salt, drought and heat stress was then investigated. The obtained results showed that all three *VaCML92*-transgenic plant lines exhibited a higher survival rate under cold stress than the control KA0 plant line (Figure 4a). The survival rate of the *VaCML92*-transgenic plants under cold stress treatment was significantly increased, by 1.8–2.2-fold (Figure 4a), as a result of the overexpression of the *VaCML92* gene. Under drought stress, the survival rate of the two plant lines with the highest *VaCML92* transgene expression (L92-I and L92-III) was significantly higher than that of the KA0 plants, indicating that *VaCML92* overexpression conferred drought resistance to *A. thaliana* (Figure 4c). At the same time, the salt and heat stress resistance of the *VaCML92*-transgenic *A. thaliana* plants was not significantly altered (Figure 4b,d).

### 2.3. VaCML92 Expression in V. amurensis Cell Cultures and Leaves after the Cold Stress

It has previously been demonstrated that the cold stress treatment did not lead to an increase in the expression of endogenous *VaCML92* in the leaves of *V. amurensis* [10]. Consequently, these findings contradicted the newly obtained results, which showed that *VaCML92* overexpression improved cell culture and plant resistance to cold stress. In the previous experiments [10], RNA was extracted after 6, 12 and 24 h of cold exposure. Plants are known to possess rapid-response genes whose expression increases sharply and rapidly upon exposure to a stress factor and then returns to normal or even to reduced levels [35].

**Figure 5:**
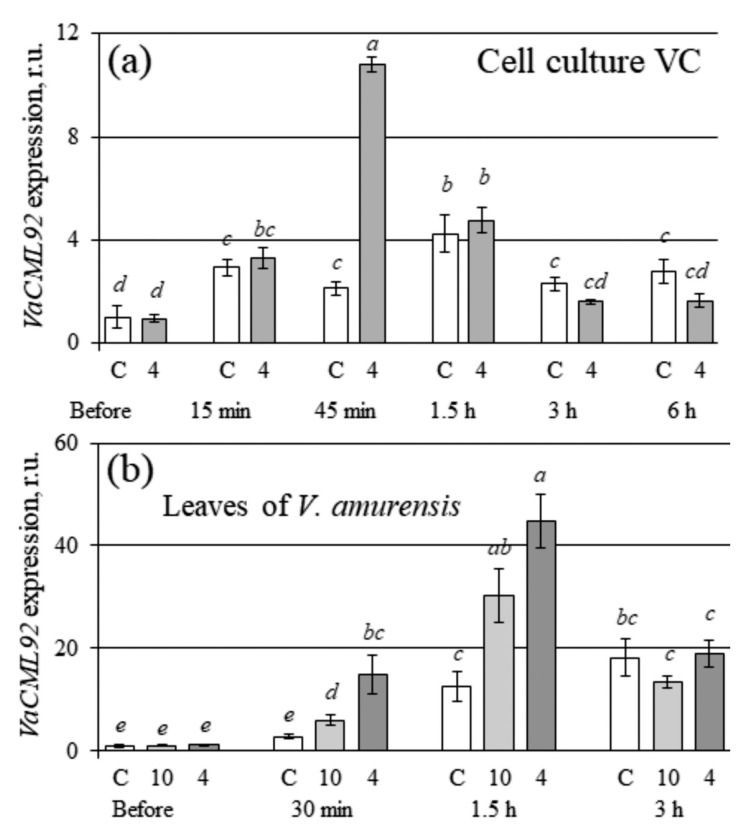
Quantification of the endogenous *VaCML92* expression in VC callus cell culture (**a**) and leaves (**b**) of *Vitis amurensis* performed through RT-qPCR. Data are presented as mean ± SE. Means followed by the same letter were not different using Student’s *t*-test. *p* < 0.05 was considered to be statistically significant.

It is proposed that the *VaCML92* gene may belong to the genes of rapid response to cold stress. To test this hypothesis, the VC control callus cell culture was subjected to cold stress (cultivation at 4 °C), and the total RNA was extracted before treatment and after 15 min, 45 min, 1.5 h, 3 h and 6 h of the cold and control treatment. It has been shown that *VaCML92* gene expression was sharply increased under cultivation at 4 °C compared to its expression before treatment, reaching the highest level after 45 min of cold exposure (Figure 5a). A considerable increase in *VaCML92* expression was also observed under the control conditions, but at a lower level. The *VaCML92* expression then decreased and remained at the same level of expression as in the cells cultured under the control conditions (Figure 5a).

The obtained results were then verified on the expression of *VaCML92* shortly after cold stress using the leaves of *V. amurensis* grapevine. For this purpose, the total RNA was extracted before the cold treatment (cultivation at 10 °C and 4 °C) and after 30 min, 1.5 h and 3 h of the cold and control treatments. The data showed a significant increase in *VaCML92* expression under both the control and cold stress conditions (Figure 5b). The up-regulation of the *VaCML92* mRNA levels was more pronounced under the cold stress conditions, with a sharp increase at 30 min and 1.5 h of the treatments (Figure 5b), whereas it decreased to the control levels after 3 h of cold exposure (Figure 5b).

### 2.4. Stilbene Biosynthesis in VaCML92-Overexpressing Cell Cultures of V. amurensis

As secondary metabolites are an essential part of the plant defense response, it is important to analyze the effect of *VaCML92* overexpression on the content of stilbenes in *V. amurensis*, particularly because stilbenes are phenolic compounds with pronounced protective properties and health-promoting activities [21,22,23].

**Figure 6:**
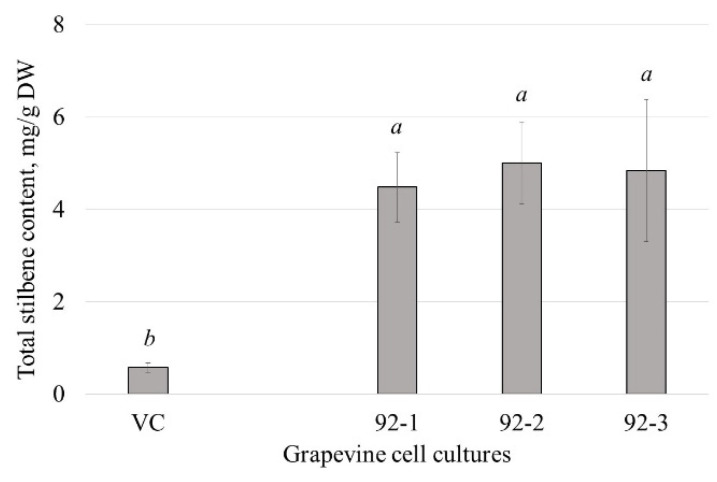
Total stilbene content in *VaCML92*-overexpressing and control *Vitis amurensis* cell lines in mg per g of the dry weight (DW). VC—control cell line transformed with the empty vector harboring only the *nptII* gene, a selective marker; 92-1, 92-2, 92-3—cell lines transformed with the *VaCML92* gene. Data are presented as mean ± SE. Means followed by the same letter were not different by Student’s *t*-test. *p* < 0.05 was considered to be statistically significant.

The HPLC analysis revealed that the total content of stilbenes in all three independently obtained grapevine cell cultures—namely, 92-1, 92-2 and 92-3—was 7.8–8.7 times higher than in the control VC culture (Figure 6). The total content of stilbenes reached 4.5–5 mg/g of the dry weight. This increase was mainly due to an increase in the content of *t*-resveratrol (17.6–20.2 times, compared to VC) and viniferins (4.3–6.7 times, compared to VC) (Table 1).

### 2.5. Expression of the Stress-Responsive Genes in Transgenic Arabidopsis Plants

**Figure 7:**
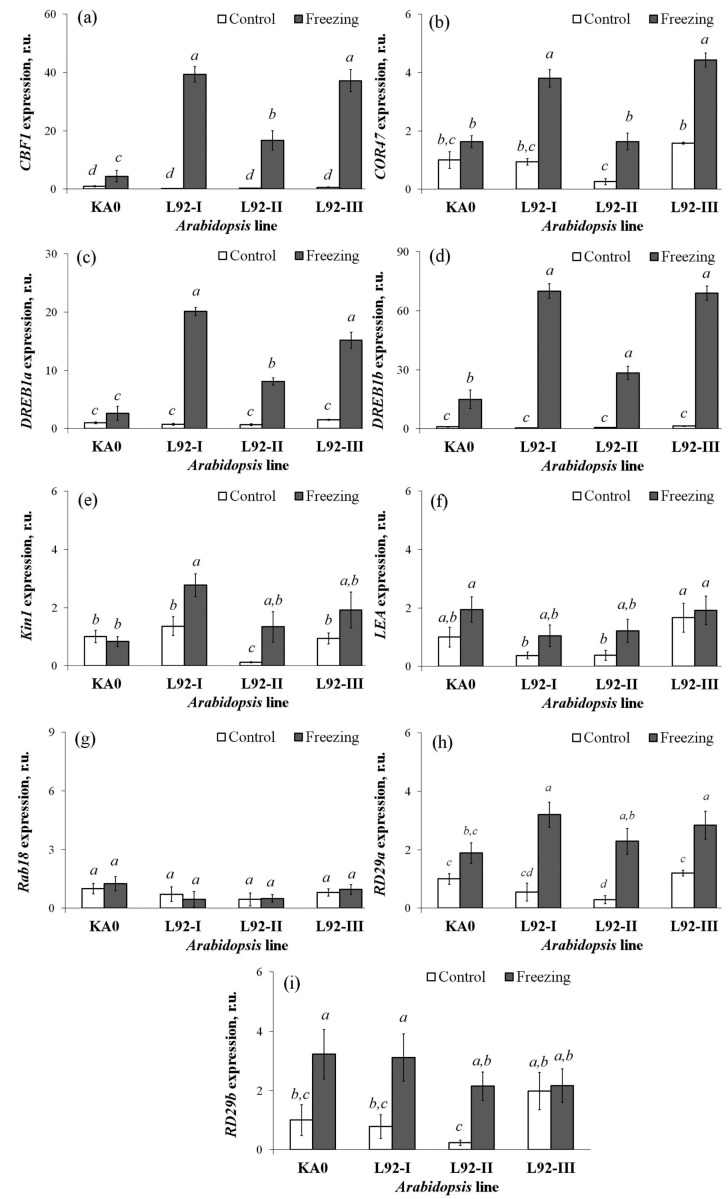
Expression of the stress-responsive genes *AtCBF1* (**a**), *AtCOR47* (**b**), *AtDREB1a* (**c**), *AtDREB1b* (**d**), *AtKIN1* (**e**), *AtLEA* (**f**), *AtRAB18* (**g**), *AtRD29a* (**h**) and *AtRD29b* (**i**) in the 3-week-old *VaCML92*-overexpressing (L92-1, L92-2 and L92-3) and vector control (VC) *Arabidopsis thaliana* exposed to control (22 °C) and cold stress conditions for 1.5 h (−10 °C). Total RNA was extracted from plants 24 h after the cold stress treatment. Data are presented as mean ± SE. Means followed by the same letter were not different using Student’s *t*-test. *p* < 0.05 was considered to be statistically significant.

To test the involvement of the *VaCML92* gene in the regulation of cold tolerance in *Arabidopsis*, the expression of a number of cold stress-related marker genes, including *AtCBF1*, *AtCOR47*, *AtDREB1a*, *AtDREB1b*, *AtKIN1*, *AtLEA*, *AtRAB18*, *AtRD29a* and *AtRD29b*, was examined (Figure 7).

Overexpression of the *VaCML92* gene resulted in a considerable activation of *AtCBF1* (Figure 7a), *AtDREB1A* (Figure 7c) and *AtDREB1b* (Figure 7d) gene expression in all three transgenic lines of *A. thaliana*. The expression of the *AtCOR47*, *AtKIN1* and *AtRD29a* genes was significantly increased in 1–2 transgenic lines (Figure 7b,e,h). At the same time, the expression of the genes *AtLEA*, *AtRAB18* and *AtRD29b* did not change significantly (Figure 7f,g,i).

## 3. Discussion

In the plant kingdom, there are five recognized families of calcium sensors; namely, *CDPK*, *CaM*, *CML*, *CBL* and *CIPK* [4,5,6]. Among these families, CDPK has received the most extensive research, demonstrating its diverse biological functions and its ability to play both positive and negative regulatory roles in the plant response to various stress conditions [37]. However, there is little information about the biological functions of other calcium sensor protein genes. Therefore, it was decided to investigate the role of the *VaCML92* gene, a member of the *CML* gene family that actively responds to abiotic stress [10]. Previously, it has been shown that *VaCML92* mRNA levels increased considerably in response to high salinity, water deficit and osmotic stress [10]. However, the present data, obtained using *VaCML92*-transgenic cell cultures of *V. amurensis* and transgenic plants of *A. thaliana*, showed no significant resistance to drought or soil salinity in the *VaCML92*-overexpressing *V. amurensis* and *A. thaliana*, while the accumulation of grapevine biomass and the survival rate of *Arabidopsis* plants were considerably improved compared to the control cells and plants. Furthermore, in contrast to the data of the present study, the previous data showed that the expression of the endogenous *VaCML92* in the leaves of *V. amurensis* did not increase under cold stress at 4 °C, and even decreased significantly at 10 °C [10]. It should be noted that RNA was extracted 6, 12 and 24 h after cold exposure in the mentioned experiments [10], while there is a likelihood that *VaCML92* could be involved in the cold stress response as a rapid response gene. A number of plant rapid response genes are known whose expression is rapidly activated after abiotic stress exposure, before their expression subsequently returns to the previous level [35]. To verify this hypothesis, the VC cell culture was subjected to cold stress (cultivation at 4 °C) and the total RNA was extracted before treatment and after 15 min, 45 min, 1.5 h, 3 h and 6 h of the cold and control treatments. It has been shown that *VaCML92* gene expression increased sharply and then gradually decreased to the previous level. The data show that *VaCML92* is a rapid cold-responsive gene.

The heterologous overexpression of the grapevine *VaCML92* gene in *A. thaliana* plants resulted in an improved plant survival rate after freezing and enhanced the expression of several cold-inducible stress marker genes (*AtCBF1*, *AtDREB1A* and *AtDREB1b*) in all three lines or in two lines (*AtCOR47* and *AtRD29a*), supporting the positive role of *VaCML92* in plant cold stress adaptation. It is known that the cold-induced CBF transcriptional regulatory factor (TF) CBF1 controls the cold-responsive expression of a large cold-responsive (COR) gene regulon that enhances plant freezing tolerance [38,39]. The *AtDREB1A* and *AtDREB1B* genes encode two stress-inducible transcription factors, DRE-BINDING PROTEIN 1A and 2A (DREB2A and DREB1A), which are known to regulate the dehydration-responsive element (DRE)-mediated transcription of target genes under cold, dehydration and high salinity stress conditions [40,41,42]. The *AtCOR47* gene encodes a dehydrin protein [43,44,45]. Dehydrins are a family of proteins found in plants that are produced in response to cold and drought stress and are known for their protective role against abiotic stress, including cold stress [44,46]. Little information is available in the literature on the physiological roles of the proteins encoded by *RD29a* [47], but it is known that the expression level of the *RD29a* gene is induced in response to salt, cold, dehydration and osmotic stress [47]. 

Previously, the involvement of *CML* genes in the biosynthesis of stilbenes was studied using inducers of stilbene biosynthesis, stilbene precursors and UV treatment [30], as these are among the most potent activators of stilbene biosynthesis [23]. However, the expression of the *VaCML92* gene either did not change or decreased under most treatments, while it increased strongly only 1 h after UV-C irradiation, and then returned to the previous levels [30]. Therefore, the data on the involvement of *VaCML92* in stilbene biosynthesis revealed that the *VaCML92* gene belongs to the rapid response genes that positively regulate stilbene production.

## 4. Materials and Methods

### 4.1. Plant Material and Used Chemicals

The parent V7 callus culture was established in 2017 from young stems of mature *V. amurensis* grapevines collected from the Primorsky Region of the Far East of Russia and identified at the Federal Scientific Center of the Biodiversity (FEBRAS), as described in [31]. Transgenic cell cultures of *V. amurensis* (VC, 92-1, L92-II, L92-III) used in the present study were obtained from the V7 cell suspension culture through agrobacterial transformation with pZP plasmids (pZP-RCS2-*VaCML92*-*nptII* or empty vector pZP-RCS2-*nptII*) [32], as described in [31]. Briefly, V7 culture *calli* (0.5 g) were transferred to a liquid modified Murashige and Skoog (MS) W_B/A_ medium [31] in 250 mL flasks and incubated at 24–25 °C in the dark on a rotary shaker. An overnight suspension of *Agrobacterium tumefaciens* GV3101 was added to a 7-day-old grape cell culture. After 2 days, 250 mg/L cefotaxime (Cf) was added. After a period of 5–7 days, the cells were moved to a fresh W_B/A_ solid medium supplemented with 250 mg/L Cf and 15 mg/L kanamycin (Km). Subsequently, during a 3-month cultivation period, the *calli* were subjected to 250 mg/L of Cf in order to control bacterial growth. The selection process for transgenic grapevine cell aggregates was carried out for a period of 3 months in the presence of 15–20 mg/L of Km.

The V7 and transgenic *calli* were cultivated with 35-day subculture intervals in the dark at 24–25 °C in test tubes (height 150 mm, internal diameter 16 mm) with 8 mL of the solid modified MS W_B/A_ medium [31] supplemented with 0.5 mg/L 6-benzylaminopurine, 2 mg/L α-naphthaleneacetic acid and 8 g/L agar.

To generate transgenic *Arabidopsis* plant lines overexpressing the *VaCML92* gene, the same plasmid constructs (pZP-RCS2-*VaCML92*-nptII, pZP-RCS2-*nptII*) were used for the overexpression of *VaCML92* in *V. amurensis* cell cultures. The overexpression construct was introduced into the *Agrobacterium tumefaciens* strain GV3101::pMP90 and transformed using the floral dip method into plants of wild-type *A. thaliana* ecotype Columbia [34]. Briefly, *Arabidopsis* inflorescences were fully immersed in a solution containing *A. tumefaciens* GV3101. Subsequently, the seeds were collected from the treated plants. Transgenic plants were selected by kanamycin (Km) resistance on half-strength MS medium supplemented with 50 μg/mL Km and confirmed through PCR using the primers for *VaCML92* and *nptII* genes, listed in Table S1. The PCR products were verified through DNA sequencing. The *Arabidopsis* transgenic lines (KA0, L92-I, L92-II, L92-III) used in this study were homozygous plants with single copy insertion. The plants were cultivated in soil under specific conditions: 22 °C under a 16/8 h day/night cycle at a light intensity of ~120 μmol m-2s-1. All chemicals used for plant calli cultivation and transformation were obtained from Sigma-Aldrich (St. Louis, MO, USA) and Himreaktivsnab (Ufa, Russia).

### 4.2. Salt, Cold and Heat Treatments of Transgenic Callus Cell Lines

For the abiotic stress treatments, the transgenic cell cultures, VC, 92-1, 92-2 and 92-3, of *V. amurensis* were cultivated in the dark at 24–25 °C in 150 × 16 mm test tubes with 8 ± 0.5 mL of the W_B/A_ medium [48]. Salt treatment was applied by adding 50 and 100 mM of NaCl to the W_B/A_ medium. Mannitol treatment (osmotic stress) was applied by adding 0.2 and 0.3 M of D-mannitol to the medium. The NaCl and D-mannitol used for the stress treatments were obtained from (Panreac AppliChem, Darmstadt, Germany) and Himreaktivsnab (Ufa, Russia), respectively. Cold and heat treatments (low- and high-temperature stress) were performed by culturing the transgenic cells at 16 and 33 °C in a growth chamber (TSO-1/80 SPU, SKTB, Smolensk, Russia) on the W_B/A_ culture media for 30 days. The average growth rates were assessed after 30 days of cultivation under control or stress conditions. The inoculum biomass in the test tube was 0.15–0.17 g (each callus was weighed using an electronic balance). Each experiment consisted of ten replicates for each cell line under the control or stress conditions.

### 4.3. Drought, Salt, Cold and Heat Treatments of Transgenic Arabidopsis Plants

Wild-type and transgenic sterilized seeds were germinated on the 1/2 MS medium solidified with 0.8% agar. Then, the seedlings were grown on the MS medium for 7 days and transferred to commercially available rich, well-watered soil in a controlled environmental chamber at 22 °C (Sanyo MLR-352), kept on a 16/8 h day/night cycle at a light intensity of ~120 µmol m^−2^ s^−1^. 

The plants were subjected to drought, salt, cold and heat treatments, as described in [48]. Briefly, the plants were subjected to drought stress treatments by culturing without additional irrigation until lethal effects were observed for all genotypes (4–5 weeks without additional irrigation) and then re-watered. For salt stress treatments, the 7-day-old seedlings were transferred to the well-watered soil and cultivated without additional irrigation for 2 weeks. Then, the plants were well-irrigated with NaCl solution (350 mM) applied at the bottom of the pots. When the soil was completely saturated with salt water, free NaCl solution was removed and the plants were cultured as normal. One week after irrigation with NaCl, the pots were placed in 3 cm deep fresh water for 4 h to leach the salt from the soil and to make morphological differences under severe salt stress more distinctive. For cold tolerance assays, normally cultured *Arabidopsis* plants (3-week-old) were stressed in a −10 °C freezer (GA-B489YVCZ, LG, Republic of Korea) for 1.5 h and then cultured at 8 °C for 2 h to facilitate recovery. For heat tolerance assays, normally cultured *Arabidopsis* plants (3-week-old) were stressed at 45 °C in a controlled incubator (ES-20/60 BioSan, Riga, Latvia) for 4 h. The survival rates were determined as the number of visibly green plants 3 days (drought) or 1 week (salt, cold and salt stresses) after rehydration. For each stress treatment, each pot had ten seedlings planted. Two pots of plants were grown for each line (genotype) and each treatment in one experiment. The experiments were repeated six times for each stress treatment type, and similar results were observed for each group of experiments.

### 4.4. Stilbene Analysis by High Performance Liquid Chromatography (HPLC) and Mass Spectrometry

Total stilbene content from the 35-day-old cell cultures was measured through HPLC, as in described [36]. The quantification of all components was carried out using two different high-performance liquid chromatography (HPLC) systems. The first system was the LC-20 analytical HPLC system manufactured by Shimadzu (Kyoto, Japan). This system consisted of an SPD-M20A photodiode array detector, LC-20ADXR pump, Shim-pack XR-ODS II column and SIL-20ACXR auto sampler. The second system used was the 1260 Infinity analytical HPLC system developed by Agilent Technologies (Santa Clara, CA, USA). This system was equipped with a G1315D photodiode array detector, G1311C quaternary pump, G1316A column oven and G1329B auto sampler. To analyze the samples, the HPLC systems were connected to a Bruker HCT ultra PTM Discovery System, which is an ion trap mass spectrometer manufactured by Bruker Daltonik GmbH in Bremen, Germany. This mass spectrometer was used for further analysis and identification of the components.

The extracts were separated on a Zorbax C18 column (150 mm, 2.1-mm i.d., 3.5-µm part size, Agilent Technologies, USA). The mobile phase consisted of a gradient elution of 0.1% aqueous acetic acid (A) and acetonitrile (B). The gradient profile with a flow rate of 0.2 mL/min was: 0 min 0% B; 35 min 40% B; 40 min 50% B; 50 min 100% B; and then eluent B until 60 min.

### 4.5. RNA Isolation, Reverse Transcription and Quantitative Expression Analysis (RT-qPCR)

Total RNA extraction was performed using the cetyltrimethylammonium bromide (CTAB)-based extraction, as described in [48]. In summary, plant leaves (0.05 g) or calluses (0.3 g) were homogenized in 1.4 mL of buffer consisting of 100 mM Tris (pH 8.0), 2 M NaCl, 25 mM EDTA (pH 8.0), 2% CTAB and 2% PVP. The homogenate was then incubated at 65 °C for 5 min using a programmable thermostat (Gnom, DNK-technology, Moscow, Russia). Following this, 500 µL of chloroform was added and the samples were centrifuged at full speed (10,000 g) for 10 min (5415R, Eppendorf, Hamburg, Germany). Subsequently, 1 mL of the aqueous phase was combined with 250 µL of 10 M lithium chloride (LiCl) and incubated overnight at 4 °C. The resulting mixture was centrifuged at full speed for 20 min. The resulting pellets were then dried at 37 °C for 30 min and reconstituted in 100 µL of distilled water. Afterwards, 300 µL of ethanol was added to the samples and incubated overnight at −20 °C. The samples were once again centrifuged at full speed for 20 min. The pellets were dried as described earlier, and finally dissolved in 100 µL of distilled water. The chemicals used for RNA isolation were obtained from Sigma-Aldrich (St. Louis, MO, USA) and Himreaktivsnab (Ufa, Russia).

Complementary DNAs were synthesized using 1.5 µg of RNA using the MMLV Reverse Transcription PCR Kit with oligo(dT)15 (Evrogen, Moscow, Russia). The RT-PCR reactions were performed in 50 µL aliquots of the reaction mixture, which contained 1x RT buffer, 1 mM each of the 4 dNTPs, 2 µM of dithiothreitol (DTT), 1 µM of oligo-(dT)15 primer, 250 U of MMLV-polymerase at 37 °C for 1.5 h. The 1 µL samples of reverse transcription products were then amplified through qPCR.

RT-qPCRs were performed using a real-time PCR kit (Evrogen, Moscow, Russia) and SYBR Green I for RT-qPCR (Evrogen, Moscow, Russia) using cDNAs, as described in [49]. cDNAs were amplified by utilizing 20 μL reaction mixtures consisting of 1× TaqMan Buffer, 2.5 mM MgCl_2_, 250 μM of each deoxynucleotide, 1 U Taq DNA polymerase, 0.5 μL (20 ng) cDNA sample, 0.25 μM of each primer and cDNA probe. The amplification procedure included an initial cycle of 2 min at 95 °C, followed by 50 cycles of 10 s at 95 °C and 25 s at 62 °C. The 2^−ΔΔCT^ method was used to calculate the expression [49]. Two internal controls were used for grapevine cells (*VaGAPDH* and *VaActin1*) [36] and for *Arabidopsis* (*AtGAPDH* and *AtUBQ*), as described in [33]. Detailed information on the genes and primers used can be found in Table S2.

### 4.6. Statistical Analysis

To study the expression of transgenes, two separate experiments were carried out, each consisting of ten technical replicates. In the first experiment, five RT-qPCR reactions were performed and normalized to the first internal control gene (*VaGAPDH* for *V. amurensis* and *AtGAPDH* for *Arabidopsis*). Similarly, in the second experiment, five RT-qPCR reactions were conducted and normalized to the second internal gene (*VaActin1* for *V. amurensis* and *AtUBQ* for *Arabidopsis*). For the analysis of callus tissue weight, three independent experiments were conducted, with ten technical replicates in each experiment. Additionally, three independent experiments were performed to analyze stilbene, with two technical replicates in each experiment. The obtained data were presented as the mean ± standard error (SE). Statistical analysis was performed using the Student’s *t*-test in the Excel software, version 1808. A *p*-value of less than 0.05 was considered statistically significant. In summary, the study involved multiple experiments and replicates to analyze transgene expression, callus tissue weight and stilbene content. The statistical significance of the results was determined using the Student’s *t*-test.

## 5. Conclusions

The results of this research indicate that the grapevine *VaCML92* gene plays a positive role in protecting plants from the detrimental effects of cold stress and, to a lesser extent, drought. This gene is rapidly activated in response to low temperature and improves the cold stress resistance of plants by activating some TFs, dehydrins and Ser/Thr protein kinases (such as CBF1, DREB1a, DREB1b, COR47, RD29). These findings have the potential to establish a theoretical basis for understanding the molecular mechanisms underlying grapevine adaptation to cold and drought. Furthermore, the *VaCML92* gene can be used in biotechnology as a gene to create cold-tolerant varieties of agricultural crops.

## Figures and Tables

**Table 1:** The content of individual stilbenes (mg/g of dry weight, DW) in the control VC cell line and *VaCML92*-overexpressing cell lines (92-1, 92-2 and 92-3) of *Vitis amurensis*. *—*p* < 0.05; **—*p* < 0.01 versus values of individual stilbene levels in the control VC cell line.

Grapevine Cell Cultures	(1) ^a^ *t*-Resveratrol Diglucoside, mg/g DW	(2) *t*-Piceid, mg/g DW	(3) *Cis*-Piceid, mg/g DW	(4) *t*-Piceatannol, mg/g DW	(5) *t*-Resveratrol, mg/g DW	(6) *Cis*-Resveratrol, mg/g DW	(7) ε-Viniferin, mg/g DW	(8) δ-Viniferin, mg/g DW
VC	0.14 ± 0.02	0.14 ± 0.02	0.01 ± 0.01	0.01 ± 0.01	0.21 ± 0.09	0.01 ± 0.01	0.01 ± 0.01	0.05 ± 0.03
92-1	0.18 ± 0.03	0.05 ± 0.02 *	0.01 ± 0.01	0.01 ± 0.01	3.87 ± 0.73 **	0.01 ± 0.01	0.11 ± 0.02 **	0.27 ± 0.03 **
92-2	0.17 ± 0.02	0.19 ± 0.08	0.01 ± 0.01	0.01 ± 0.01	4.43 ± 0.82 **	0.01 ± 0.01	0.07 ± 0.02 *	0.14 ± 0.02 *
92-3	0.16 ± 0.03	0.08 ± 0.03	0.01 ± 0.01	0.01 ± 0.01	4.31 ± 1.34 *	0.01 ± 0.01	0.09 ± 0.02 **	0.21 ± 0.05 **

^a^—the numbers of stilbene compounds in parentheses correspond to the numbers of stilbenes presented on the HPLC chromatogram, Kiselev et al. 2023 [36].

## Data Availability

The data presented in this study are available within the article and Supplementary Materials.

## Supplementary Materials

The following are available online at https://www.mdpi.com/article/10.3390/ijms242115827/s1.
