# Supplementary material for: Involvement of the Calmodulin-like Protein Gene VaCML92 in Grapevine Abiotic Stress Response and Stilbene Production
Source: Int J Mol Sci. 2023 Oct 31;24(21):15827. doi: 10.3390/ijms242115827 (PMC10649675; doi:10.3390/ijms242115827)
Supplement: Supplementary file 1 [file ijms-24-15827-s001.zip › ijms-2648031-supplementary.pdf]

International Journal of *Molecular Sciences*

MDPI

Supplementary Materials

Involvement of the Calmodulin-like Protein Gene *VaCML92* in Grapevine Abiotic Stress Response and Stilbene Production

Olga A. Aleynova, Konstantin V. Kiselev, Andrey R. Suprun, Alexey A. Ananev and Alexandra S. Dubrovina *

Laboratory of Biotechnology, Federal Scientific Center of the East Asia Terrestrial Biodiversity, Far Eastern Branch of the Russian Academy of Sciences, 690022 Vladivostok, Russia; kiselev@biosoil.ru (K.V.K.)

* Correspondence: dubrovina@biosoil.ru; Tel.: +8-423-2310410; Fax: +8-4232-310193

Citation: Aleynova, O.A.; Kiselev, K.V.; Suprun, A.R.; Ananev, A.A.; Dubrovina, A.S. Involvement of the Calmodulin-like Protein Gene *VaCML92* in Grapevine Abiotic Stress Response and Stilbene Production. *Int. J. Mol. Sci.* **2023**, *24*, 15827. https://doi.org/10.3390/ijms242115827

Academic Editor: Fabrizio Grassi

Received: 20 September 2023
Revised: 27 October 2023
Accepted: 30 October 2023
Published: 31 October 2023

Copyright: © 2023 by the authors. Licensee MDPI, Basel, Switzerland. This article is an open access article distributed under the terms and conditions of the Creative Commons Attribution (CC BY) license (https://creativecommons.org/licenses/by/4.0/).

Table S1. Primers used for amplification of *Arabidopsis thaliana* and *Vitis amurensis* cDNAs in PCR.

cDNA	Primers names	Primers sequences, 5'-3'
**Primers for obtaining of the full *VaCML92* cDNA and generation the construction for plant cell transformation**		
Full *VaCML92* cDNA obtaining	VaCML92-nachS<br>VaCML92-KonA	5'ATGAGAAGAGAAGATACAGCA,<br>5'CTAGCGCATCATAGCGGA
Overexpression of *VaCML92*	VaCML92-Hind-pSATs<br>VaCML92-Bam-pSATa	5'GCTCAAGCTTATGAGAAGAGAAGATACAGC<br>5'TCGAGGATCCCTAGCGCATCATAGCGGAGA
**Primers for real-time PCRs in *A. thaliana* plants and grapevine *V. amurensis* cell cultures**		
Overexpression of *VaCML92* transgenes	VaCML92-real-S<br>pSAT-term-A	5'AACGGAGATGGCGTCCTC,<br>5'GAGAGACTGGTGATTTTTGCG
Total overexpression of *VaCML92* gene	VaCML92-real-S<br>VaCML92-Kon-A	5'AACGGAGATGGCGTCCTC,<br>5'CTAGCGCATCATAGCGGA
Overexpression of *VaCML92* endogen	VaCML92-real-S<br>VaCML92-real-endo-A	5'AACGGAGATGGCGTCCTC,<br>5'TGGCTGTGGGTGATGAAGAAG
VaActin1 (DQ517935)	*VaActin*-realS<br>*VaActin*-realA	5'GTATTGTGCTGGATTCTGGTGA,<br>5'GCAAGGTCAAGACGAAGGATAG
VaGAPDH (AM437491)	*VaGAPDH*-realS<br>*VaGAPDH*-realA	5'CACTGAAGATGATGTTGTTTCC,<br>5'GCTATTCCAGCCTTGGCAT
AtEF (XM_002864638)	AtEF-1a-OT-s<br>AtEF-1a-OT-a	5'TGCCCCTGGACATCGTGATT,<br>5'CTTGGGCTCGTTGATCTGGT
AtGAPDH (NM_111283.4)	AtGapdh-Real-s<br>AtGapdh-Real-a	5'TTGGTGACAACAGGTCAAGCA,<br>5'AAACTTGTCGCTCAATGCAAT
**Primers for semiquantitative RT-PCR**		
*npt*II (AY818371)	NPTII-SER-S<br>NPTII-KON-A	5'ATTCGACCACCAAGCGAAAC,<br>5'TCAGAAGAACTCGTCAAGAA
VirB2 (KY000054)	VIRB2-S<br>VIRB2-A	5'ATGCGATGCTTTGAAAGATACCG,<br>5'TTAGCCACCTCCAGTCAGCG
